# Assessment of health literacy among older Finns

**DOI:** 10.1007/s40520-018-1104-9

**Published:** 2018-12-21

**Authors:** Johanna Eronen, Leena Paakkari, Erja Portegijs, Milla Saajanaho, Taina Rantanen

**Affiliations:** 1 Gerontology Research Center, Faculty of Sport and Health Sciences, University of Jyväskylä, P.O. Box 35, 40014 Jyvaskyla, Finland; 2 Research Center for Health Promotion, Faculty of Sport and Health Sciences, University of Jyväskylä, Jyvaskyla, Finland

**Keywords:** Aging, Health literacy, Health, Functioning, Cross-sectional study

## Abstract

**Aims:**

This study examined the feasibility of the HLS-EU-Q16 (in Finnish) for use among older Finns and whether the health literacy score correlates with indicators of health and functioning.

**Methods:**

To determine the feasibility of the instrument, we first conducted a focus group discussion with nine participants. For the quantitative analyses, we used data from the AGNES cohort study, collected between October 2017 and April 2018 at the University of Jyväskylä in Finland. 292 75-year-old Finnish men and women were interviewed face-to-face in their homes. Health literacy was measured with the HLS-EU-Q16 and health literacy score, ranging from 0 to 50, computed. The reproducibility of the instrument was test-retested. Chi-square tests were used to compare health literacy scores between participants by different socioeconomic variables, and Spearman correlation coefficients were calculated to study the associations of health literacy with cognition, depressive symptoms, chronic conditions, life-space mobility and physical performance.

**Results:**

The mean health literacy score for all participants was 35.05 (SD 6.32). Participants who rated their financial situation and self-rated health as very good had the highest health literacy scores (38.85, SD 5.09 and 39.22, SD 6.77, respectively). Better health literacy was associated with better cognitive status, fewer depressive symptoms and chronic conditions, higher life-space mobility and better physical performance.

**Conclusions:**

The HLS-EU-Q16 is a feasible measure for research purposes among older Finns. The associations between health literacy and indicators of health and functioning need to be more closely investigated in larger samples with a wider age-range.

## Background

Health literacy means the competence to access, understand, appraise and apply health information to be able to make decisions concerning one’s health care, disease prevention and health promotion [1]. This definition views health literacy from the public health perspective as a broad concept which encompasses an individual’s understanding of and ability to use health information and ability to navigate a complex health care system [2].

Health literacy is a critical determinant of health [3]. Among older people low health literacy has been associated with unfavorable health behaviors and health outcomes, such as poorer compliance with physical activity guidelines [4], poorer adherence [5], higher prevalence of chronic conditions [6], poorer cognition [7], and a higher number of doctor visits [8]. In addition, health literacy is an important factor contributing to autonomy and empowerment [3]. Good health literacy enables older people to make independent health decisions and engage in the activities that promote and sustain health, whereas low health literacy may weaken their autonomy, and hence limit their independence, freedom and dignity.

In a recent German study, 66% of people over the age of 65 and 80% of those over 76 had limited health literacy [9], compared to roughly 50% in the general population [10]. The higher proportion of older people with low health literacy supports the claim that health literacy presents a challenge for public health in Europe [10]. Moreover, the level of health literacy is not only the lowest in the older age groups [8, 11, 12], but is also not equally distributed within this population segment. Studies of several indicators, such as education, income and perceived social status, have reported that higher socioeconomic status is associated with better health literacy [6, 9]. At the same time, many older individuals have at least one illness or chronic condition that requires self-care, including coping with medication and access to or contact with health care personnel [13, 14]. Consequently, older adults, and especially those in the lower social strata may be the most vulnerable in terms of limited health literacy [10, 15]. When this finding is combined with the fact that poorer cognitive functioning and strong cognitive decline during late adulthood are associated with low health literacy [7], older adults are at greater risk for a mismatch between the competencies needed to take care of health and everyday challenges that they face.

Despite increasing interest in the field, major gaps in the literature remain. To gain a better understanding of the role of health literacy in the lives of older adults, we need more information on the determinants and correlates of health literacy in this population. For example, the role of cognition [7, 16] and morbidity [17] as determinants of health literacy warrant further examination. In addition, research on health literacy in older persons should be extended from physical and mental health to include other components of wellbeing. Opportunities to participate in meaningful activities and maintain social connections are important for wellbeing in old age [18]. Opportunities may depend on the individual’s functional capacity or mobility in their environment, that is, life-space mobility, which can shape the degree to which one is able to make and act on health-related choices and decisions [19, 20]. Moreover, information on the association of health literacy with different health behaviors, such as physical activity and exercise, would be of value in efforts to promote healthy lifestyle in old age [21].

The importance of assessing health literacy among older adults has been recognized [6, 9, 22]. Both objective (e.g. REALM, TOFHLA [15]) and subjective (e.g. HLQ [23]) measures have been applied in studies conducted among older persons. While objective measures mostly focus on literacy and numeracy, the use of more comprehensive instruments that focus on older adults’ self-perceptions of their task-specific competencies in various health domains have become more popular in the field of public health during recent years. The European Health Literacy Survey (HLS-EU) is a comprehensive measure which includes the domains of health care, disease prevention and health promotion and has been used in various populations. It was therefore chosen to assess older persons’ health literacy in this study.

To the present authors’ best knowledge, this is the first study to apply the HLS-EU-Q16 questionnaire among older Finns. Thus, the first aim was to examine the feasibility and psychometric properties of the HSL-EU-Q16 questionnaire in the target population by evaluating its test–retest reproducibility and face validity. We then examined the distribution of the health literacy score according to various socioeconomic and personal factors and examined the correlations of health literacy with cognitive capacity, depressive symptoms, chronic conditions, life-space mobility and physical performance.

## Methods

### Participants

Participants for the focus group discussion were recruited through the University of the Third Age in Jyväskylä. Six women and three men aged 66–89 years formed the group. Finnish was the native language of the participants. The discussion took place in February 2018.

For the quantitative analyses, we used data, collected between October 2017 and April 2018 at the University of Jyväskylä, Finland, on the 75-year-old participants in the “Active aging—resilience and external support as modifiers of the disablement outcome (AGNES)” cohort study. Details of the AGNES cohort study have been published earlier [24]. Briefly, the AGNES cohort study comprises a population-based sample of men and women aged 75, 80 and 85 residing in the city of Jyväskylä in Central Finland. Those living independently in the study area, able to communicate, and willing to participate took part in the AGNES study. The present analyses comprised 292 75-year-old men and women. After an initial invitation and phone interview, the eligible participants filled out a postal questionnaire and, typically within 1 week, were interviewed in their homes by trained interviewers using Computer Assisted Personal Interview. To test the reproducibility of the instrument, 18 people responded to the HLS-EU-Q16 a second time approximately 1 week after the initial interview.

The ethical committee of the Central Finland Health Care District gave ethical approval to the AGNES study protocol on August 23, 2017. All participants signed an informed consent before entering the study. The principles of the Declaration of Helsinki were followed throughout the study.

### Focus group interview

The focus group interview was held at a university facility and lasted 2 h. First, the participants reflected on their overall impression of the questionnaire and the relevance of each item to them, the discussion proceeding from item to item in linear order. The analysis is based on the field notes taken by the two interviewers present during the discussion. The discussion was not audiotaped. The interviewers’ notes were combined and read through several times. Based on their similarities and differences, the notes were then grouped to describe participants’ opinions about the instrument.

### Quantitative measurements

Health literacy was measured with the Finnish translation of the European Health Literacy Survey Questionnaire 16 (HLS-EU-Q16) [2]. The instrument was first translated from English into Finnish and then back-translated by two different professional translators. According to the guidelines provided by the developers of the HLS-EU-Q, we calculated a health literacy score only for participants who had answered at least 80% of the items, i.e. given answers to at least 13 items in the questionnaire [10]. The score was calculated using the following formula:

$${\text{Index}}=\left( {{\text{mean}} - 1} \right) \times \left( {\frac{{50}}{3}} \right).$$

This formula yields a score ranging from 0 to 50, where 0 is the lowest and 50 the best possible health literacy score. Scores can then be divided into four quartiles: 0–25 indicating inadequate, > 25 to 33 problematic, > 33 to 42 sufficient and > 42 to 50 excellent health literacy [10].

Sex was derived from the population register as part of the initial sampling process; all the other demographic variables were derived from the AGNES postal questionnaire. Participants were asked to report their marital status (married or cohabiting vs. unmarried or divorced vs. widowed), whether they were living alone or with someone, and home ownership (own, partially owned or rented). Educational level was assessed by asking the participants to report their highest educational attainment, which was categorized as low (primary school or less), medium (middle school, folk high school, vocational school or secondary school), or high (high school diploma or university degree). Occupational status was elicited by asking the participants to report their longest-held occupation and their most recent occupation. These were classified according to Statistics Finland’s Classification of Occupations and the one ranked higher in the classification was taken as representing the participant’s occupation and further categorized into upper non-manual, lower non-manual or manual occupations [25]. Participants were asked to rate their self-perceived financial situation on a four-point scale from very good to poor and to report whether they acted as a family caregiver. Self-rated health was assessed with a question about the general health of the participant on a rating scale from very good, to very poor. Due to the low number of responses in the poor and very poor health categories, these were combined for the further analyses.

In the home interview, cognitive capacity was assessed with the Mini-Mental State Examination (MMSE) [26] and depressive symptoms with the Center for Epidemiologic Studies Depression Scale (CES-D) [27]. MMSE scores range from 0 to 30 with higher scores indicating better cognition; CES-D scores range from 0 to 40 with higher scores indicating more depressive symptoms. Self-reported physician-diagnosed chronic conditions were elicited during the home interview and the total number of different chronic conditions (respiratory, cardiac, vascular, cerebrovascular, neurological and musculoskeletal conditions, visual or auditory impairments, diabetes mellitus, malignant cancer and depression) was calculated. Life-space mobility was assessed with the University of Alabama at Birmingham Study of Aging Life-Space Assessment (LSA) [20]. The assessment comprises 15 items asking about ability to move on six life-space levels (bedroom, other rooms, outside home, neighborhood, town, beyond town). Participants were asked how many days per week they had attained each level during the past 4 weeks and whether they had needed assistive devices or help from another person. The life-space mobility composite score, indicating distance, frequency and level of independence, ranges from 0 to 120, with higher scores indicating higher life-space mobility. Physical performance was measured with the Short Physical Performance Battery (SPPB) [28]. The SPPB score ranges from 0 to 12, higher scores indicating better physical performance.

### Statistical analyses

The test–retest reliability of the health literacy scale was determined by calculating intra-class correlation (ICC) coefficient. Kendal’s Tau B was used to analyze agreement between the individual items. The internal consistency of the HLS-EU-Q16 scale was measured with Cronbach’s alpha for the total sample. The distribution of the answers to the 16 items is reported as frequencies and percentages. Health literacy scores by the personal and socioeconomic variables are presented as means, standard deviations (SD) and ranges. Differences in the mean scores between categories were tested with analyses of variance (ANOVA). Correlations between indicators of health and functioning and health literacy score were estimated with Spearman correlation coefficients. All analyses were performed with IBM SPSS version 24.

## Results

### Focus group interview

Overall, the focus group members reported that all items in the questionnaire were easy to understand. There were no misunderstandings and the contents of the questions were understood as intended. The questions were also relevant to the participants of the focus group. Everyone had experiences of navigating the health care system, the concept of health-related information was clear, and the conversation reflected the participants’ skills in identifying the potential sources of health-related information.

### Test–retest

Among the 18 persons who took part in the retest, the mean health literacy score obtained from the home interview was 35.9 (SD 5.9) and from the retest 35.4 (SD 5.5). The intra-class correlation coefficient for these data over the 1-week interval was 0.782, *p* < 0.001. Kendal’s Tau B correlation for the individual items between test and retest are presented in Table 1. In seven out of sixteen questions, the *p* value for the correlation coefficient was smaller than 0.05, indicating similarity in the participants’ responses to those items on both occasions.

**Table 1:** Test–retest

	Kendal’s Tau B coefficient	*p* value
Find information on treatments of illnesses that concern you	**0.453**	**0.044**
Find out where to get professional help when you are ill	− 0.168	0.478
Understand what your doctor says to you	**0.620**	**0.007**
Understand your doctor’s or pharmacist’s instruction on how to take a prescribed medicine	0.172	0.475
Judge when you may need to get a second opinion from another doctor	**0.475**	**0.028**
Use information the doctor gives you to make decisions about your illness	**0.468**	**0.049**
Follow instructions from your doctor or pharmacist	0.249	0.294
Find information on how to manage mental health problems like stress or depression	0.437	0.056
Understand health warnings about behavior such as smoking, low PA and drinking too much	0.438	0.071
Understand why you need health screenings	0.357	0.141
Judge if the information on health risks in the media is reliable	**0.580**	**0.010**
Decide how you can protect yourself from illness based on information in the media	0.198	0.376
Find out about activities that are good for your mental well-being	**0.589**	**0.008**
Understand advice on health from family members or friends	**0.715**	**0.001**
Understand information in the media on how to get healthier	0.321	0.134
Judge which every day behavior is related to your health	0.385	0.113

Bold values indicate statistically significant result, i.e. *p* < 0.05

Kendal’s Tau B coefficients and *p* values. Includes only participants with complete information on both occasions (*n* = 18)

### Descriptive analyses

Information about health literacy was available for 292 participants, of whom 57.1% were women. The mean health literacy score across all participants was 35.05, standard deviation (SD) 6.32. 4.8% of the participants had inadequate, 31.5% problematic, 51.4% sufficient and 12.3% excellent health literacy.

Cronbach’s Alpha for the HLS-EU-Q16 questionnaire was 0.87. The distributions of answers to the 16 items are shown in Table 2. Items that were most often rated as “very easy” were understanding health warnings about behavior (Q9; 78.1%), understanding why one needs health screenings (Q10; 68.8%), understanding instructions on taking prescribed medication (Q4; 57.5%) and instructions from doctors/pharmacists (Q7; 49.8%). All three questions which dealt with information in the media (Q11, Q12, Q15) were the ones most often rated as “fairly difficult” (by 48.2%, 47.5% and 40.5%, respectively).

**Table 2:** Distributions of answers to the HLS-EU-Q16 items

On a scale from very easy to very difficult, how easy would you say it is to …	Very difficult	Fairly difficult	Fairly easy	Very easy	Missing
	*n* (%)	*n* (%)	*n* (%)	*n* (%)	*n* (%)
1. Find information on treatment of illnesses that concern you?	10 (3.3)	40 (13.3)	171 (56.8)	72 (23.9)	8 (2.7)
2. Find out where to get professional help when you are ill?	2 (0.7)	34 (11.3)	154 (51.2)	103 (34.2)	8 (2.7)
3. Understand what your doctor says to you?	2 (0.7)	28 (9.3)	164 (54.5)	99 (32.9)	8 (2.7)
4. Understand your doctor’s or pharmacist’s instruction on how to take a prescribed medicine?	0	8 (2.7)	112 (37.2)	173 (57.5)	8 (2.7)
5. Judge when you may need to get a second opinion from another doctor?	12 (4.0)	112 (37.2)	125 (41.5)	38 (12.6)	14 (4.7)
6. Use information the doctor gives you to make decisions about your illness?	4 (1.3)	24 (8.0)	161 (53.5)	102 (33.9)	10 (3.3)
7. Follow instructions from your doctor or pharmacist?	0	8 (2.7)	135 (44.9)	150 (49.8)	8 (2.7)
8. Find information on how to manage mental health problems like stress or depression?	6 (2.0)	56 (18.6)	171 (56.8)	59 (19.6)	9 (3.0)
9. Understand health warnings about behavior such as smoking, low physical activity and drinking too much?	0	3 (1.0)	55 (18.3)	235 (78.1)	8 (2.7)
10. Understand why you need health screenings?	0	4 (1.3)	90 (29.9)	198 (65.8)	9 (3.0)
11. Judge if the information on the health risks in the media is reliable?	8 (2.7)	145 (48.2)	116 (38.5)	23 (7.6)	9 (3.0)
12. Decide how you can protect yourself from illness based on information in the media?	19 (6.3)	143 (47.5)	109 (36.2)	22 (7.3)	8 (2.7)
13. Find out about activities that are good for your mental well-being?	2 (0.7)	69 (22.9)	166 (55.1)	54 (17.9)	10 (3.3)
14. Understand advice on health from family members or friends?	4 (1.3)	47 (15.6)	164 (54.5)	76 (25.2)	10 (3.3)
15. Understand information in the media on how to get healthier?	22 (7.3)	122 (40.5)	118 (39.2)	30 (10.0)	9 (3.0)
16. Judge which everyday behavior is related to your health?	0	20 (6.6)	176 (58.5)	96 (31.9)	9 (3.0)

Gender and socioeconomic factors were not systematically associated with the health literacy score except for perceived financial situation which showed a positive association (Table 3). Favorable values in indicators of health and functioning such as self-rated health, depressive symptoms, cognitive capacity, physical performance and life-space mobility were associated with better health literacy (Tables 3, 4).

**Table 3:** Health literacy score means, standard deviations (SDs) and ranges by personal and socioeconomic variables

	%	Mean	SD	Range	*p* value
Gender (*n* = 292)					0.975
Women	57	35.06	6.25	20.83–50.00	
Men	43	35.03	6.44	16.63–50.00	
Marital status (*n* = 291)					0.852
Unmarried/divorced	17	34.64	6.27	15.63–46.88	
Married/cohabiting	68	35.19	6.34	16.67–50.00	
Widow/widower	16	34.91	6.47	22.92–50.00	
Living situation (*n* = 292)					0.683
Alone	32	34.83	6.70	15.63–50.00	
With someone	68	35.15	6.15	18.75–50.00	
Home ownership (*n* = 291)					0.981
Own	87	35.08	6.12	16.67–50.00	
Rental	7	34.97	7.26	15.63–47.92	
Partly owned	6	34.77	8.60	18.75–47.92	
Educational level (*n* = 292)					0.066
High	32	36.30	6.61	18.75–50.00	
Medium	51	34.39	6.40	15.63–50.00	
Low	17	34.69	5.22	26.04–47.92	
Occupational status (*n* = 274)					0.541
Upper non-manual	40	35.16	6.32	34.38–50.00	
Lower non-manual	37	34.79	6.78	16.67–50.00	
Manual	23	34.06	6.36	23.96–50.00	
Perceived financial situation (*n* = 291)					**0.005**
Very good	9	38.85	5.09	30.21–50.00	
Good	54	34.72	6.40	15.63–50.00	
Satisfactory/poor	37	34.63	6.23	22.92–50.00	
Family caregiver (*n* = 277)					
No	86	34.93	5.67	15.63–50.00	0.583
Yes	14	35.47	6.50	22.92–44.79	
Self-rated health (*n* = 292)					< **0.001**
Very good	6	39.22	6.77	38.54–50.00	
Good	49	35.94	6.16	21.88–50.00	
Moderate	41	33.83	5.94	16.67–47.92	
Poor/very poor	4	30.73	6.70	15.63–39.58	

Bold values indicate statistically significant difference between categories, i.e. *p* < 0.05

**Table 4:** Means and standard deviations (SDs) of indicators of health and functioning, and their bivariate correlations with health literacy score

	Range	Mean	SD	Correlation coefficient	*p* value
Cognitive status (MMSE)	16–30	27.61	2.12	0.125	0.034
Depressive symptoms (CES-D)	0–40	7.84	6.53	− 0.342	< 0.001
Number of chronic conditions	0–7	2.65	1.52	− 0.277	< 0.001
Life-space mobility	6–110	74.11	17.59	0.236	< 0.001
Physical performance (SPPB)	0–12	10.52	1.91	0.235	< 0.001

## Discussion

The HLS-EU-Q16 instrument proved a feasible method for investigating health literacy in older Finnish individuals. The participants found the questionnaire items relevant and understandable, and the questionnaire was easy to administer as part of a larger study. The repeatability of the individual items in the retest was fair, with dissimilar responses to nine items; however, the health literacy score means did not differ between the two test occasions.

Our findings suggest that in this population, health literacy correlates with indicators of health and functioning: older persons with better health literacy had better self-rated health and cognition, reported fewer depressive symptoms and chronic conditions and had greater life-space mobility and better physical performance. These results indicate that in old age health literacy is a resource that may have an effect in preventing age-related deteriorations in health and functioning. While some of our results confirm those of earlier studies [6, 9], the novelty of this study was the inclusion of both subjective (e.g. life-space mobility, self-rated health) and objective (e.g. physical performance, cognition) measures of functioning, which are informative about older persons abilities to carry out daily activities [18, 29]. The observed associations justify closer scrutiny of the relationship of health literacy and old age functioning in future studies.

The participants with the highest education showed the highest health literacy scores. However, this association was not statistically significant. Previous studies have reported mixed findings on the association between education and health literacy in older adults [6, 30]; this is understandable, as many older adults have acquired education in early adulthood and some circumstances later in life, such as occupation during working age, may have a stronger influence than early formal education. However, we found no association with occupation or home ownership, either. On the other hand, in line with some previous studies [10, 30], participants whose perceived financial situation were very good also had the highest health literacy scores. These findings suggest that in studies conducted among the older population, several different indicators of socioeconomic position should be included to help identify those who are at higher risk for poorer health due to low health literacy [10].

In the present sample of 75-year-olds, cognitive capacity, measured with the MMSE, was associated with health literacy. This is in line with a recent study, which showed that poorer cognitive functioning is associated with lower health literacy [7]. However, it should be noted that the majority of our participants had relatively good cognitive capacity. To better understand the complex association between health literacy and cognition, more research among older-olds is needed.

### Strengths and limitations

Strength of the study was the use of a validated measure of health literacy in a population-based sample. The HLS-EU-Q16 instrument, administered as part of a larger study covering a number of different subjective and objective measures, allowed us to examine associations between health literacy and indicators of functioning that have not previously been utilized. Quantitative data were collected with face-to-face interviews, which enabled the interviewers to assist participants if they had problems with their vision. The focus group interview findings supported the use of the HLS-EU-Q16 instrument among older people.

This study has its limitations. First, we used the short version instead of the full version of the HLS-EU-Q. Second, our study sample contained only75-year-old native Finns whose first language was Finnish, and therefore we cannot generalize our findings to all older persons, or those with migrant background. Third, the study was cross-sectional, and thus the associations found should not be understood as causal. Finally, due to the diversity of measures of health literacy used in previous studies, our results are not fully comparable to those of studies that have applied different measures.

## Conclusion

This study yielded novel information on the role of health literacy in health and functioning among older persons. We found the HLS-EU-Q16 to be a feasible measure for use in older populations and that enabled us to find correlations between health literacy and indicators of health and functioning. These preliminary findings on the role of health literacy in health and functioning in old age lay a foundation for more detailed analyses with different study designs.
